# Upregulation of miR-330-5p is associated with carotid plaque’s stability by targeting Talin-1 in symptomatic carotid stenosis patients

**DOI:** 10.1186/s12872-019-1120-5

**Published:** 2019-06-18

**Authors:** Xiaolong Wei, Yudong Sun, Tonglei Han, Jiang Zhu, Yongfu Xie, Shiying Wang, Yani Wu, Yinxing Fan, Xiuli Sun, Jian Zhou, Zhiqing Zhao, Zaiping Jing

**Affiliations:** 1 Department of Vascular Surgery, Changhai Hospital, Second Military Medical University, 168 Changhai Road, Shanghai, 200433 China; 2 Depaertment of general surgery, Nanjing General Hospital of Eastern Theater Command, Nanjing, China; 3 Department of Breast and Thyroid Surgery, Changhai Hospital, Second Military Medical University, Shanghai, China; 4 Zhenjiang Medical District, General Hospital of Eastern Theater Command, Zhenjiang, China; 5 Department of ophthalmology, Jinan aier eye hospital, Jinan, China

**Keywords:** Talin-1, Carotid artery stenosis, Plaque, Stability, miR-330

## Abstract

**Background:**

The aim of this study was to investigate the relationship between Talin-1 and stability of carotid atherosclerosis plaque and also find out the role of miRNA, as an upstream regulator, in regulating the expression level of Talin-1.

**Methods:**

Human carotid plaques were obtained from 20 symptomatic carotid stenosis patients who underwent carotid endarterectomy (CEA) in our hospital between October 2014 and August 2017. Western blot analysis and immunohistochemistry was carried out to detect the distribution and expression level of Talin-1 in each plaque sample. The content of miRNAs in carotid plaque was decected by quantitative reverse transcription polymerase chain reaction (RT-qPCR), and the relative expression levels were calculated by 2^-△△Ct^ method after the (cycle threshold) Ct value (power amplification knee point) was obtained. Dual-luciferase reporter assays were applied to verify the successful transfections. Finally, we compared all the groups with independent-samples t-test and one-way analysis of variance (ANOVA).

**Results:**

Talin-1 was significantly downregulated in human unstable carotid plaque samples compared with stable carotid plaques (*P* < 0.05), and the distribution of Talin-1 was mainly found in the fibrous cap of carotid plaque. The overexpression of miRNA-330-5p was found in unstable carotid plaque, which significantly induced the inhibition of expression level of Talin-1.

**Conclusion:**

Upregulated miR-330-5p may lead to unstable carotid plaques by targeting Talin-1 in symptomatic carotid stenosis patients. This might be a new target for the treatment of atherosclerotic diseases through future studies.

## Background

Stroke is the second most frequent cause of death and the third leading reason of long-term physical disability in humans [1, 2]. The proportion of atherosclerotic carotid artery stenosis has risen to 20% of all the etiological factor of ischemic stroke worldwide [1]. At present, clinical topical issue in carotid stenosis disease is mainly concentrated on the identification of the stability of plaque. Plaque stability is related to the integrity of the fibrous cap, the morphology of the necrotic core, the function of endothelial cells, and smooth muscle cells [3–6]. Any insignificant change may break the balance. Thrombus formation after plaque rupture can cause myocardial infarction, stroke, morbidity, and even death, and these obvious evidences associate with the development and prognosis of carotid atherosclerotic disease, which may be directly affected by the stability of atherosclerosis plaque [3, 7, 8].

Talin-1, which has been reported as an essential mediator of integrin activation, can deeply influence the integrin crosstalk, associating with cell adhesion, migration, anoikis, survival, and cytoskeleton remodeling [9–14]. Recent studies have revealed that Talin-1 plays a substantial role in the metastasis process of tumor cells [15–17], influencing cell proliferation by recruiting and activating focal adhesion proteins, as well as influencing an association of integrin adhesions with cell cycle progression [18]. Furthermore, these regulatory functions are often mentioned in the process of plaque formation and stability, and could stimulate us to find out some studies concerning the relationship of Talin-1 with plaque stability. To date, a limited number of studies have assessed the relationship of plaque stability with Talin-1. It has been reported that Talin-1 was dramatically down-regulated in atherosclerotic samples compared with normal tissues [19]. However, the relationship between Talin-1 and carotid artery plaque stability is still elusive.

In addition, miRNA is a class of approximately 20~22 nucleotides long, short noncoding RNA molecules, known by the capable of inhibiting target gene expression as posttranscriptional negative regulators [20, 21]. The overexpression of miRNAs, as an a potential biomarker and modulator, has caused a great advance in experimentally detecting and therapeutically regulating their expression level. Based on human cardiovascular system’s specific ability to sensitive subtle gene expression alterations, miRNAs might be excellent drug targets for atherosclerotic disease [22]. Previous studies have confirmed some particular miRNAs, which are related to the stability of atherosclerosis plaques, such as miRNA-210, miRNA-155, and miRNA-33 [3, 23, 24]. If miRNAs participate in regulating carotid arteryplaque stability needs to be further studied.

In our research, we hypothesized that miRNA-330-5p and expression level of Talin-1 in carotid artery showed significant difference between stable and unstable plaques. Thus, we further detected the regulatory effect of miRNA-330-5p on Talin-1 which might play a substantial role in plaque stability in carotid artery.

## Methods

### Patients’ recruitment and sample collection

Human carotid plaques were obtained from twenty symptomatic carotid stenosis patients who underwent carotid endarterectomy (CEA) in the Department of Vascular Surgery of Changhai Hospital (Shanghai, China) between October 2014 and August 2017. Informed consent was obtained from each subject prior to start of the research. The indication for surgery was > 70% asymptomatic or > 50% symptomatic carotid stenosis. Our study only enrolled symptomatic patients’ sample. If patients had recently a TIA or ischemic stroke in the vascular territory supplied by a moderate or severe carotid artery stenosis or carotid occlusion within the preceding 4 weeks (early phase), and symptoms were attributed to the carotid artery stenosis, those were considered as symptomatic carotid stenosis patients. We also excluded patients with Ehlers-Danlos syndrome, bicuspid aortic valves, Marfan syndrome, and connective tissue disease, in addition to those who were refused to participate in the study. Every sample of arterial tissue was collected within 30 min after CEA. In order to wipe off the blood and mural thrombus adhering to the vascular wall, every specimen was flushed with precooled saline solution for at least five times. By using sterile tweezers and eye scissors, the samples were all sliced up as roughly 0.5 cm pieces along longitudinal axis in a clean Petri dish immediately. Then, all the tissue samples were into two parts that one part was sliced up as approximately 2 mm and placed it in sterile EP tubes, and the other part fixed in 4% paraformaldehyde and then embedded in paraffin. Tissues in the former part were then frozen in liquid nitrogen fleetly and stored at − 80 °C until analysis, and the process was accomplished within 10 min. As the tissues in the latter part were inflicted, no accessional surgery beyond the standard care was required to obtain these additional specimens. Table 1 presents the demographic and clinical characteristics of the patients and controls. The research was conducted in accordance with the 1964 Declaration of Helsinki, and approved by the Ethic Committee of Changhai Hospital.

**Table 1:** Demographic and clinical data of symptomatic carotid stenosis patients

	Stable Plaque (n = 10)	Unstable Plaque (n = 10)
Age, years	64.10 ± 5.20	65.70 ± 6.70
Sex, male:female	6:4	6:4
Hypertension, n (%)	7 (70.00)	5 (50.00)
Hyperlipidemia, n (%)	3 (30.00)	4 (40.00)
Diabetes mellitus, n (%)	1 (10.00)	0 (0.00)
Coronary artery disease, n (%)	0 (0.00)	0 (0.00)
Previous MI, n (%)	0 (0.00)	0 (0.00)
Previous Stroke, n (%)	2 (20.00)	2 (20.00)
Body mass index, kg/m^2^	24.52 ± 3.16	23.51 ± 2.40
Carotid Stenosis, (%)	84.60 ± 7.06	82.70 ± 7.09
Current smoking, n (%)	4 (40.00)	3 (30.00)
Alcohol consumption, n (%)	2 (20.00)	1 (10.00)
Previous or ongoing drug treatment, n (%)		
Antihypertensive drugs	3 (30.00)	5 (50.00)
Hypoglycemic drugs	1 (10.00)	0 (0.00)
Statins	0 (0.00)	0 (0.00)
Antiplatelet drugs	0 (0.00)	0 (0.00)
TG, mmol/L	1.18 ± 0.41	1.47 ± 0.60
HDL, mmol/L	1.10 ± 0.12	1.12 ± 0.13
LDL, mmol/L	1.70 ± 0.28	1.84 ± 0.35
Creatinine, mg/dL	71.10 ± 18.52	65.40 ± 17.40
eGFR, ml/min/1.73m^2^	75.47 ± 42.68	82.93 ± 26.01

### Clinical measurement

Patients’systolic blood pressure (SBP) and diastolic blood pressure (DBP) was measured in three intervals at rest by using a mercury sphygmomanometer. Standardized interview questionnaires were used to collect participants‘information which included the demographic and lifestyle characteristics. Each patient was asked about the smoking status, current drug use and past disease history. Hypertension was defined as SBP ≥ 140 mmHg and/or DBP ≥ 90 mmHg. Hyperlipidemia was defined as low-density lipoprotein cholesterol (LDL-C) ≥ 3.5 mmol/L and/or total cholesterol (TC)/high-density lipoprotein cholesterol (HDL-C) ≥ 5 and/or use of prescribed lipid-lowering medications at the time of the health check. Coronary artery disease was defined via patients who suffered from the diseases, such as stable angina, unstable angina, or myocardial infarction. Based on the usual ethanol intake: liquor (40%), sake (16%), wine (12.5%) and beer (5%), the monthly alcohol consumption and grams of ethanol per day was calculated. The patients with alcohol consumption were defined as average daily ethanol consumption more than 5 g.

### Carotid plaques’ histological assessment

Hematoxylin and eosin (H&E) staining was carried out to exhibit the morphological characteristics of the plaques on serial sections of each sample according to the classification defined by the American Heart Association (AHA) [25]. The following were identified as stable plaques: Type I lesion contains scattered macrophage foam cells; Type II lesion have formed fatty streaks; Type III lesions contain dispersed aggregates of extracellular lipid droplets and granules which destroy the coherence of some intimal smooth muscle cells; Type IV was defined as plaque contains a larger, confluent, and more disruptive core of extracellular lipid; Type Va was defined as plaque with fibrous connective tissue together with extracellular lipids and laminated acellular collagen without endothelial disruption; type Vb was defined as the plaque with only fibrous conjunctive tissue; Type VIa was defined as the stable lesions sorted into AHA type V, meanwhile, ulceration of the endothelial surface; Type VIb was defined as the stable lesions sorted into AHA type V; Type VIc was defined as plaque with intraplaque thrombosis. The classification of carotid artery lesions was undertaken by two independent observers. These carotid plaque samples collected from patients underwent carotid endarterectomy were always type IV, type V or type VI.

### Western blotting

Through the use of 1000 mL of RIPA buffer, total protein was extracted from 100 mg tissue standardizing all the specimens to 1.0 mg/ml. On a 6 to 12% sodium dodecyl sulfate polyacrylamide gel electrophoresis plate, a total of 20 mL was added and transferred onto a polyvinyllidenedifluoride (PVDF) membrane, thus each sample’s total amount was 20 μg based on the of Western blotting. Blocking the membranes with 5% nonfat milk (Becton Dickinson, Franklin Lakes, NJ, USA), as well as washing and exploring with the following primary antibodies: rat anti-human Talin-1 (1:1000; Abcam, Cambridge, UK), and GAPDH (1:500; Abcam, Cambridge, UK). After washing, membranes were incubated with a horseradish peroxidase (HRP)-conjugated goat anti-rat (1:2000; Sigma-Aldrich, St. Louis, MO, USA) for 1 h. Then, on the basis manufacturer’s instructions (Thermo Fisher Scientific, Waltham, MA, USA), membranes were washed and cultivated using an enhanced chemiluminescence kit. Each sample was analyzed in triplicate by Western blotting. Afterwards, Image-Pro Plus 6.0 software (Media Cybernetics, Inc., Rockville, MD, USA) was applied to analyze the band intensities using a flatbed scanner.

### Immunofluorescence (IF) staining

The IF staining for Talin-1 was undertaken on 5 um thickness paraffin-embedded sections from human carotid plaque samples which were mentioned in “Patient recruitment and sample collection”. Paraffin-embedded sections were washed with phosphate-buffered saline (PBS), and then blocked by nonimmune serum for 1 h at room temperature, followed by incubation with primary antibodies (Talin-1, 1:500; Cell Signaling Technology, Danvers, MA, USA) overnight at 4 °C. After washing with PBS, the paraffin-embedded sections were incubated with a 1:200 dilution of Alexa Fluor 594 donkey anti-rabbit lgG conjugated secondary antibody (Life Technologies, Carlsbad, CA, USA) at room temperature for 30 min which were used for the visualization of signals. Nuclear staining was performed by incubating with 0.1 μg/ml DAPI (4′,6-diamidino-2-phenylindole) in the blocking solution. Semi-quantitative analyses of positive signals in samples were undertaken using Image-Pro Plus 6.0 software (Media Cybernetics, Inc., Rockville, MD, USA).

### RNA isolation and quantitative reverse transcription polymerase chain reaction (qRT-PCR)

According to the manufacturer’s instructions, total RNA was extracted from these carotid plaques by using the miRNease Mini Kit (Qiagen, Hilden, Germany) to determine the expression levels of Talin-1 and miRNAs. PrimeScript RT reagent Kit (Qiagen, Hilden, Germany) was carried out with 500 ng of total RNA to synthesize complementary DNA. Primer 5.0 software was applied to design primers (shown in Table 2) according to these published gene sequences in GenBank, and they were checked by NCBI Blast and oligo 7. Then the LightCycler 480 SYBR Green I Master (Roche Life Science, Penzberg, Germany) was employed to detect expressions of Talin-1 and miRNAs. Every SYBR Green reactionincluded each primer at 0.25 um and 2 ul of cDNA as template with a total volume of 20ul. The reactions were first incubated at 95 °C for 30 s which followed by 40 cycles at 95 °C for 5 s, at 55 °C for 10 s, and at 72 °C for 15 s. To verify that only one RT-qPCR product was detected by SYBR Green I, we thermally decomposed the samples according to the heat dissociation protocol after the last cycle of RT-qPCR to check the presence of a single peak. The expression levels of these unique miRNAs were normalized to RNU6B. After the (cycle threshold) Ct value (power amplification knee point) was achieved, we used 2^-△△Ct^ method to calculate the relative expression levels. All of these experiments were repeated three times.

**Table 2:** The sequence of primers, RNA oligo in this study

	Sequence (5′ to 3′)
Primers	
hsa-miR-6721-5p	Forward: GATTGGGCAGGGGCTTATTG Reverse: CAGTGCAGGGTCCGAGGTAT
hsa-miR-20a-3p	Forward: CGGAGACTGCATTATGAGCAC Reverse: CAGTGCAGGGTCCGAGGTAT
hsa-miR-330-5p	Forward: GCCTCTCTGGGCCTGTGTC Reverse: CAGTGCAGGGTCCGAGGTAT
hsa-miR-326	Forward: CATCGCCTCTGAGCCCTTC Reverse: CAGTGCAGGGTCCGAGGTAT
hsa-miR-4527	Forward: AGGTGGTCTGCAAAGAGATGA
	Reverse: CAGTGCAGGGTCCGAGGTAT
hsa-miR-6503-5p	Forward: GCGAGGTCTGCATTCAAATCC
	Reverse: CAGTGCAGGGTCCGAGGTAT
hsa-miR-432-5p	Forward: GGCTCTTGGAGTAGGTCATTGG
	Reverse: CAGTGCAGGGTCCGAGGTAT
hsa-miR-7150	Forward: GGTAGACTGGCAGGGGGAG
	Reverse: CAGTGCAGGGTCCGAGGTAT
hsa-miR-4260	Forward: GCAGACTTGGGGCATGGA
	Reverse: CAGTGCAGGGTCCGAGGTAT
U6B	Forward: CGCTTCGGCAGCACATATAC
	Reverse: AAAATATGGAACGCTTCACGA
RNA oligo	
miR-330-5p mimics	AAGCUGCCAGUUGAAGAACUGU ACAGUUCUUCAACUGGCAGCUU
miR-330-5p mimics NC	GCAACCUGUUAGACUAUGGAGA UCUCCAUAGUCUAACAGGUUGC
Talin-1	GUCCAUCAUUCAUGCGAAATT UUUCGCAUGAAUGAUGGACTT
Negative control	UUCUCCGAACGUGUCACGUTT ACGUGACACGUUCGGAGAATT
GAPDH Positive control	UGACCUCAACUACAUGGUUTT AACCAUGUAGUUGAGGUCATT

### Dual-luciferase reporter assays

GenePharma Co. Ltd. (Shanghai, China) synthesized the miRNA mimics, and then, the sequences of miR-330-5p mimics were exhibited in Table 2. There were 24-well plates, co-transfected with 200 ng psiCHECK-2 vector, containing 3′ UTR of Bcl-2 or 3′ UTR of Bid and 40 nM miRNA mimics/well where HEK 293 T cells were cultured. By using Lipofectamine 2000, transfections were undertaken. According to the manufacturer’s protocol, the luciferase analysis was implemented 24 h later using Dual-Luciferase Reporter Assay (Promega, Madison, WI, USA). After normalizing to renilla luciferase activity, the relative firefly luciferase activity was acquired.

### Statistical analysis

All data were presented as means (±standard deviation. SD). SPSS 17.0 software (IBM, Armonk, NY, USA) was used to perform analyses.. Independent-samples t-test and one way ANOVA analyses were utilized for determining the statistical significance between different groups. Statistically significance was considered at *P* < 0.05.

## Results

### Differential expression of Talin-1 protein in stable carotid plaque versus unstable carotid plaque

**Fig. 1:**
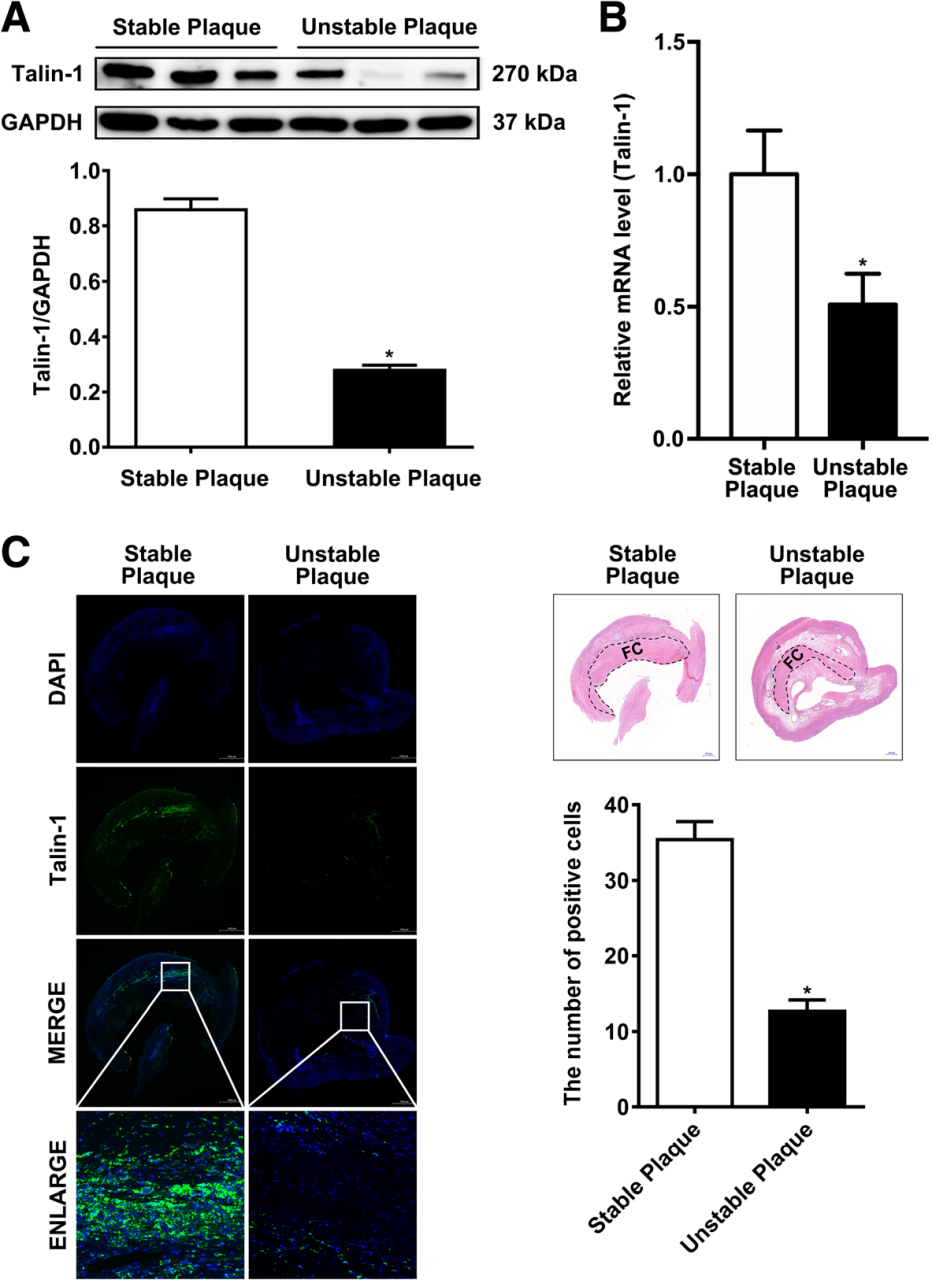
Talin-1 is downregulated in the fibrous cap of unstable carotid plaque. **a** Western blot for Talin-1 expression in human carotid plaque (*n* = 3 per group). Talin-1 was significantly downregulated in unstable carotid plaque (*P* < 0.05). **b** qRT-PCR for Talin-1 expression in human carotid plaque (*n* = 10 per group). Talin-1 was significantly downregulated in unstable carotid plaque (P < 0.05). **c** Immunofluorescent on the same sections (*n* = 3) of carotid plaque with Talin-1 staining (*n* = 10 per group). Quantitative analysis of the results of immunofluorescent revealed that Talin-1 is distributed in the fibrous cap of carotid plaque and its content was significantly downregulated in unstable carotid plaque (*P* < 0.05). Data are represented as mean ± SD. *n* = 6 randomly chosen fields in each group

Patients who underwent CEA due to symptomatic carotid artery disease were selected based on symptoms. In order to reveal the expression levels of Talin-1 in human carotid plaque, Western blot analysis was performed to detect Talin-1’s expression levels in stable and unstable carotid plaques (Fig. 1a). The expression level of Talin-1 in stable carotid plaque was significantly higher than that in unstable one. Results of Western blotting revealed that Talin-1 in unstable carotid plaque was 32.42% of stable carotid plaque. We also employed RT-qPCR to detect the mRNA level of Talin-1 in carotid plaque, in which the results showed the same findings as Western blotting (Fig. 1b). Then, immunofluorescence (IF) was performed to further detect the content and distribution of Talin-1 in human carotid plaque. As shown in Fig. 1c, the content of Talin-1 was significantly decreased in unstable carotid plaque compared with the stable carotid plaque (P<0.05), and it mainly existed in the fibrous cap of carotid plaque. These results suggest that Talin-1 showed a downregulation manner in unstable carotid plaque, especially in the fibrous cap of carotid plaque compared with stable carotid plaque.

### MiR-330-5p is significantly upregulated in unstable carotid plaques of patients with carotid stenosis

**Fig. 2:**
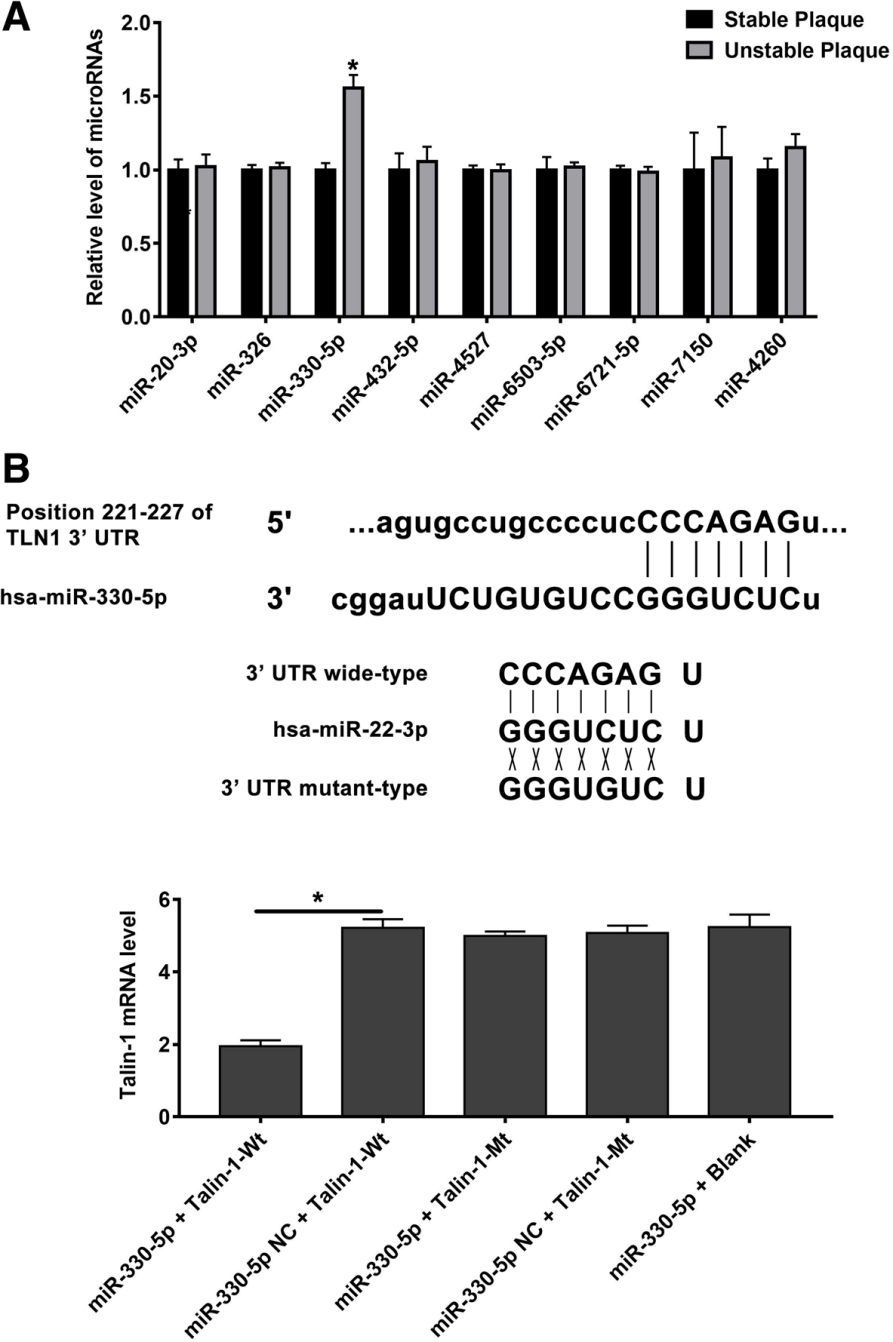
miR-330-5p is upregulated in unstable carotid plaque and Talin-1 is targeted by miR-330-5p. **a** qRT-PCR detected miRNAs expression in human carotid plaque (*n* = 10 per group). MiR-330-5p is one of the nine miRNAs which significantly upregulated in unstable carotid plaque (P < 0.05). **b** Schematic representation of the binding between miR-330-5p and Talin-1 with mutated sites labeled with gray shading. **c** The effect of miR-330-5p on Talin-1 luciferase activity by dual-luciferase assay. Data are represented as mean ± SD

The TargetScan database was used to search for miRNAs which might directly regulate the expression of Talin-1. According to the analysis, we selected 9 miRNAs to achieve further experiments. Through RT-qPCR analysis of RNAs which were extracted from carotid plaque, miR-330-5p, miR-432-5p, miR-7150, and miR-4260 were up-regulated in unstable carotid plaque. Besides, miR-330-5p was the most remarkable miRNA among the four up-regulated miRNAs in unstable carotid plaque that showed significant statistical difference compared with stable carotid plaque (Fig. 2a).

### MiR-330-5p directly targets Talin-1 mRNA

Talin-1 is a potential downstream target of miR-330-5p, while it has not yet been strongly confirmed. To indicate whether Talin-1 is a direct target of miR-330-5p, we carried out a 3’UTR luciferase analysis.. The dual luciferase reporter plasmid was designed to contain wild type or mutated miR-330-5p binding sites in the fragment of the 3’UTR of Talin-1. HEK293 cells were transfected either with miR-330-5p mimic, luciferase reporters or an unrelated miRNA as the negative control. As illustrated in Fig. 2b, Luciferase reporter analysis revealed that miR-330-5p significantly downregulated the activity of reporter which contained wild type 3’UTR of Talin-1. Mutating the complementary binding site of miR-330-5p in Talin-1 3’UTR abolished this suppression. The results demonstrated that Talin-1 is a direct target regulated by miR-330-5p.

## Discussion

In this study, by detecting the content of Talin-1 in stable and unstable atherosclerosis plaque samples, we found that the low-expression of Talin-1 may result in instability of plaque. Additionally, our outcomes revealed that Talin-1 is directly down-regulated by an upstream regulator, miRNA-330-5p, which was notably high expressed in unstable plaques. These observations suggest that cells in atherosclerosis plaque after being stimulated by some unclear factors can initiate the high-expression of miRNA-330-5p, which may inhibit synthesis of Talin-1, and eventually lead to the instability of atherosclerosis plaque.

However, to our knowledge, there have been no reports concerning the relationship between Talin-1 and carotid plaque stability. In previous studies, Talin-1 has been approved to be significant in platelet adhesion and aggregation at sites of vascular injury, and the deficiency in Talin-1 would lead to severe hemostatic defects and arterial thrombosis resistant [26]. This might be an important cause of intra-plaque hemorrhage, resulting in the instability of the plaque. In addition, the Talin-1 was indicated to play a pivotal role in enhancing the cell adhesion, cell proliferation, and angiogenesis in previous tumor relevant reports [16, 27–29]. To our knowledge, the thickness of fiber cap is a critical factor affecting the stability of the plaque, meanwhile, the ability of cell proliferation and angiogenesis may be attenuated due to the down-regulated Talin-1 in unstable plaques, and the fiber cap may become more fragile and easier to rupture. Although the possible mechanism and pathway have still remained elusive, our research has a certain guiding significance that can alter the stability of atherosclerotic plaques by changing the expression of Talin-1, acting on the formation or integrity of fibrous cap. This research also raised a new direction for further exploration of the principle of morphological changes in plaque and the mechanism of the development of plaque destabilization,

On the other hand, it is important to note that miRNA-330-5p has been demonstrated to play a vital role in the development of unstable atherosclerosis plaques, especially the effects on the endothelial proliferation through mitogen-activated protein kinase (MAPK) and WNT signaling pathways [30]. In the present study, we found that miRNA-330-5p was significantly expressed in unstable plaques, and this noticeable discrepancy may be caused by changes in the upstream regulation mechanism. As an objective of this study, the negative regulatory influence of miRNA-330-5p on the expression of Talin-1 was confirmed by using a specific siRNA, and the concentration of Talin-1 was notably decreased in siRNA transfected cells due to high expression level of miRNA-330-5p. However, it is essential to further indicate whether a decrease in the expression level of Talin-1 by interfering with the function of miRNA-330-5p can lead to changes in the stability of the plaque.

If Talin-1 can highly change the stability of the plaque through some pathways, and a number of new drug targets need to be provided for stabilizing the atherosclerosis plaque.

## Conclusions

In summary, this novel study revealed the association between Talin-1 and carotid plaque stability of symptomatic carotid stenosis patients, in which low expression level of Talin-1 may directly lead to the instability of plaques. Moreover, miRNA-330-5p, as an upstream regulator, can significantly affect the expression level of Talin-1. However, our findings should be verified through further experimental tests.

### Limitations

The present study contains some limitations. First, the current research only described a possible relationship between Talin-1 and the stability of plaque, and there are no systematic experiments in vitro and in vivo to confirm the correlation between them. Second, the stability of carotid atherosclerosis plaque owns a complicated mechanism, and although Talin-1 plays a substantial role in the regulation of plaque stability, we cannot ignore the influences of other molecular and biological factors.

## Data Availability

The datasets used and analysed during the current study are available from the corresponding author on reasonable request.

## Abbreviations

AHA: American Heart Association
CEA: Carotid endarterectomy
HE: Hematoxylin-and-eosin
